# Investigation of Solution Behavior of Antidepressant Imipramine Hydrochloride Drug and Non-Ionic Surfactant Mixture: Experimental and Theoretical Study

**DOI:** 10.3390/polym13224025

**Published:** 2021-11-21

**Authors:** Malik Abdul Rub, Naved Azum, Dileep Kumar, Muhammad Nadeem Arshad, Anish Khan, Maha Moteb Alotaibi, Abdullah M. Asiri

**Affiliations:** 1 Center of Excellence for Advanced Materials Research, King Abdulaziz University, Jeddah 21589, Saudi Arabia; nhassan2@kau.edu.sa (N.A.); mnarshad@kau.edu.sa (M.N.A.); anishkhan97@gmail.com (A.K.); aasiri2@kau.edu.sa (A.M.A.); 2 Chemistry Department, Faculty of Science, King Abdulaziz University, Jeddah 21589, Saudi Arabia; mmsalotaibi@kau.edu.sa; 3 Division of Computational Physics, Institute for Computational Science, Ton Duc Thang University, Ho Chi Minh City 700000, Vietnam; 4 Faculty of Applied Sciences, Ton Duc Thang University, Ho Chi Minh City 700000, Vietnam

**Keywords:** imipramine hydrochloride, tensiometry, mixed micelles, interaction parameter, micellar composition

## Abstract

In this paper, the interaction of imipramine hydrochloride (IMP, antidepressant drug) and a non-ionic surfactant Triton X-100 (TX-100) mixture in five different ratios through the tensiometric method in different solvents (aqueous/0.050 mol·kg^−1^ aqueous NaCl/0.250 mol·kg^−1^ aqueous urea (U)) were examined thoroughly at a temperature of 298 K. UV–Visible studies in an aqueous system of IMP + TX-100 mixtures were also investigated and discussed in detail. The pure (IMP and TX-100) along with the mixtures’ critical micelle concentration (*cmc*) were assessed by a tensiometric technique. The obtained deviation of the mixtures’ *cmc* values from their ideal values revealed the nonideal behavior of IMP + TX-100 mixtures amongst IMP and TX-100. Compared to aqueous systems, in the presence of aqueous NaCl, several changes in micelles/mixed micelles occurred, and hence a synergism/attractive interaction amongst components was found increased while in the existence of U, the synergism/attractive interaction between them decreased. The evaluated interaction parameter (*β*^Rb^) value of mixed micelles showed the attractive or synergism between the IMP and TX-100. Various evaluated thermodynamic parameters in an aqueous system showed that the mixed micellization of the IMP + TX-100 mixture was an entropically spontaneous phenomenon, although the existence of salt in all studied systems can somewhat increase the spontaneity of the micellization process and in the aqueous U system, the spontaneity of the micellization process decreased. In an aqueous system, the interaction between IMP and TX-100 was also confirmed by UV–Visible study.

## 1. Introduction

Usually, amphiphilic compounds undergo self-association into certain solvents to a rich diversity of phases liable upon their concentration as well as the solution environments, for instance, pH, temperature, and additive concentration, and so on [1,2,3,4,5]. The assembly of amphiphiles is called micelles and they have many applications in various fields such as materials synthesis, detergency, solubilization, pharmaceutical formulations, and so on [1,6,7,8]. The efficiency of surfactant implication for a certain utilization depends on their interfacial activity as well as solution mechanical/physical parameters of the associates’ structure, for example, size, shape, micellar inter-molecular interactions, and so on [9,10]. Hence, it is important to identify the basic as well as dynamic parameters of the amphiphile systems under needed experimental situations for a specific function. Despite the implication of a singular surfactant for any particular application, almost in all cases, a mixture of surfactants has been broadly used because the mixed system works show much improved properties compared with singular constituents [1,11,12]. The properties of amphiphile mixtures are generally vulnerable to the interaction amongst amphiphile monomers. These amphiphile mixtures, consisting of outstanding properties, are generally concerning the synergistic interaction amongst employed constituents, for example, the binary mixed system of ionic–nonionic, ionic–ionic molecules [1,13,14,15]. Mahajan and Nandni [16] investigated the micellization and phase behavior of binary mixtures of anionic (sodium alkyl phenol ether sulfate/sodiumbis(2-ethylhexyl) sulfosuccinate) and nonionic (polyoxyethylene alkyl ether surfactants) surfactants in aqueous media and found synergistic interactions between the constituents. Sanan and Mahajan [17] studied the interactions between mixed micelles of nonionic surfactant such as Tween and cationic surfactants such as dodecylbenzyldimethylammonium chloride and detected that the *cmc* of cationic surfactant decreased in the presence of Tween due to attractive interaction amongst employed components, as Tween monomers are intercalated into the cationic micelles.

**Figure 1:**
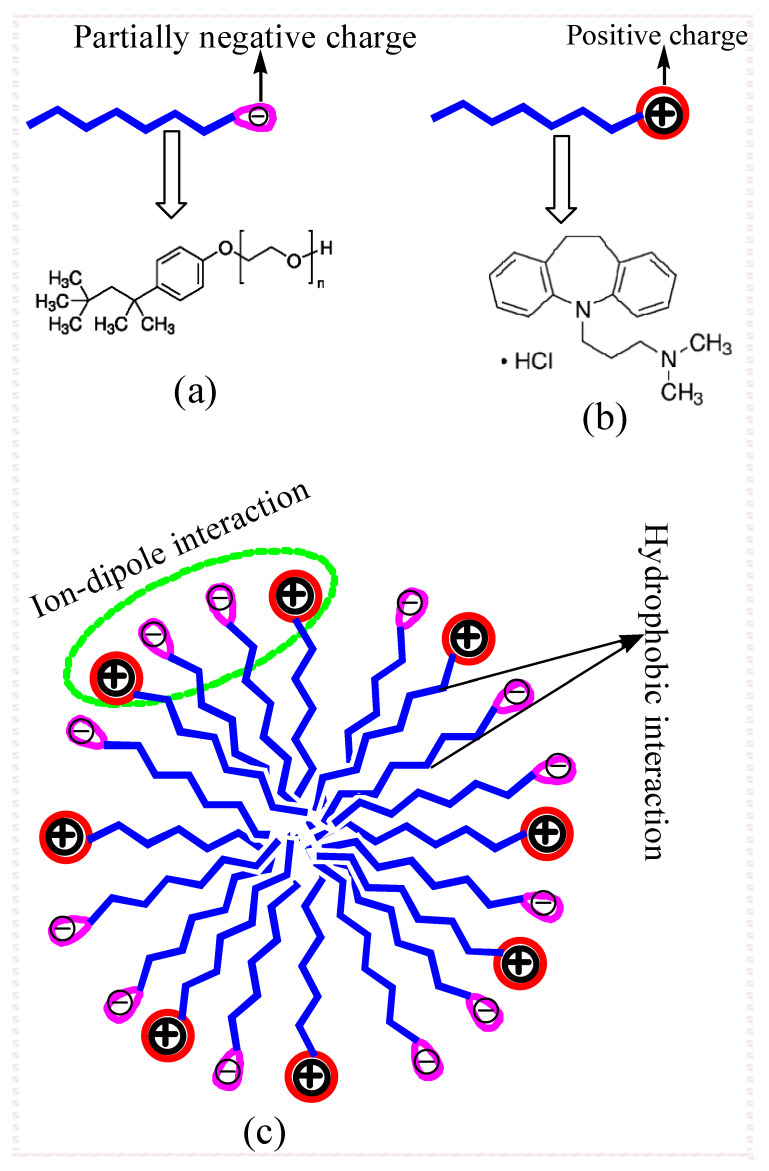
Molecular structure of (**a**) TX-100 (*n* = 9–10), (**b**) imipramine hydrochloride (IMP), and (**c**) their interaction in mixed micelles.

Among the different types of surfactants, nonionic surfactants are respected alike as perfect candidates for the sheltered conveyance of the medication, not seeing wide weakening in blood as they are physiologically progressively tolerable in contrast with ionic surfactants and, attributable to their second-rate estimations of critical micelle concentration (*cmc*) along with nonionic surfactant, are found to be less toxic compared with ionic surfactants [18,19]. Non-ionic surfactants consist of polar head groups that are not electrically charged, and these types of surfactants are usually water-soluble by means of the formation of hydrogen bonding of hydrophilic fractions through the water. Here, in the current study, Triton X-100 (TX-100) (Figure 1a) was employed to examine their interaction with the amphiphilic drug imipramine hydrochloride (IMP) (Figure 1b) in three different mediums. TX-100 was found to be clear viscous fluid in their pure form as a result of H-bonding of their hydrophilic polyethylene oxide fractions with water. TX-100 has found numerous uses in pharmaceutical commerce as well as for cleaning and as a constituent in a number of remedial products [20]. Prior to now, TX-100 has been utilized as a carrier in numerous commercially available influenza vaccines such as flublok, fluarix, and fluzone.

Similar to the usual surfactants, a number of amphiphilic drugs have also formed associate structures after crossing a certain concentration in an aqueous and nonaqueous system called micelles, but at a much higher concentration [21,22,23,24]. However, the self-association of singular amphiphilic drugs has been unfocused because usually their *cmc* were found to be too high, therefore, a large amount of drug was needed for any certain applications that may cause several side effects (due to high dose) as well as being cost-effective compared with amphiphilic drugs and additive (surfactant, bile salts, hydrotropes, etc.) mixtures that form mixed associates (mixed micelles). Amphiphilic drug and additive mixtures have been found to show much higher efficiency, much lower *cmc*, and high surface activity along with the mixture needing a very small amount for particular purposes compared with a singular system [25]. Therefore, mixed micellar solutions are used to upsurge the absorption of various drugs in human beings [21]. Imipramine hydrochloride (IMP) is an amphiphilic antidepressant drug and its structure contains a tricyclic ring core and alkylamine side chain (Figure 1b). This category of drug undergoes self-association in a somewhat similar manner to the usual surfactant because of the presence of an alkylamine side chain [21,26]. IMP is used to treat depression. This drug also shows numerous side effects such as faintness, vomiting, sleepiness, and annoyance as well as weight loss/gain, and these unwanted side effects will possibly lessen if the IMP is appropriately marked to the organism through the assistance of a drug carrier. Surfactants, especially non-ionic surfactants such as TX-100, may be one alternative as a drug carrier. Various inorganic salts and U are found in human beings and their presence can alter their micellization activity, henceforth, information of their results on the association behavior of pure component micelles along with mixed system micelles will grant the exceptional design of impressive remedial agents. Moreover, at lower concentrations, most of the drugs exhibit their pharmacological results, so it is therefore probable that the occurrence of additives such as electrolytes might reduce their *cmc* value and, thus start aggregation at a lower concentration, which may prove fatal. Therefore, in the presence of additives, the study of micellization and mixed micellization is also necessary.

As an extension of our interest in drug and surfactant mixed system interactions [27,28,29,30], herein, we investigated the interactions of IMP drug through the non-ionic surfactant TX-100 in five different ratios mixtures using the tensiometric method in aqueous, 0.050 mol·kg^−1^ NaCl, and 0.250 urea (U) medium. Earlier Alam and Siddiq [31] investigated the micellization and interfacial behavior of an IMP and Triton X-100 mixture of varying mole fraction of IMP in an aqueous system only. Mahajan et al. [32] studied the association and solution surface interactions of nortriptyline hydrochloride along with the amitriptyline hydrochloride drug with non-ionic polyoxyethylene alkyl ether surfactants by means of different techniques and they proposed a synergy between the drug and surfactant mixture having enhanced surface properties that can be proficiently used for pharmaceutical applications. Srivastava et al. [33] evaluated different parameters of the drug and anionic and non-ionic surfactant mixture micelles and employed a mixed system to improve the solubility along with a decrease in the cytotoxicity of poorly water-soluble pharmaceutical ingredients. However, up until now, according to our information, the aggregation of the IMP drug in the presence of TX-100 of various mole fraction in non-aqueous system (salt, urea) has not been stated elsewhere. This would possibly show as pharmaceutically significant in terms of unwanted side effects of the drug, which could be minimized if employed in the form of mixed micelles. Interactions of amphiphilic drug and non-ionic surfactant conceivably pictured as an alikeness for their interactions through the biomembrane, as a result, delivering an understanding of the mechanism for a more complex biological system, for example, the passing of drugs via cell membranes. The nature of IMP is amphiphilic in nature, therefore, their interaction with TX-100 is explored in the outline of hypotheses operated for a surfactant-surfactant mixed system. Numerous hypothetical models are utilized to describe the formation of the mixed micelles of IMP + TX-100 mixtures [34,35]. Regardless of the tensiometry technique, UV–Visible analysis was also operated to check the consistency in the interaction of the IMP drug with TX-100 in the aqueous system as a means to gain several basic pieces of evidence regarding the nature of interactions amongst the employed components [5,33,35].

## 2. Experimental Section

### 2.1. Materials

Each employed chemical was of analytical grade and treated for solution preparation as collected from the company with no further purification. Imipramine hydrochloride (IMP) drug and TX-100 were purchased from Sigma, St. Louis, Missouri, USA (≥98.0% (purity)) and Sigma, Taufkichen, Germany, respectively. Inorganic salt-NaCl and urea were purchased from BDH, Poole, England (98.0% (purity)), Sigma, Taufkichen, Germany (98.0% (purity)), respectively. Deionized H_2_O was employed for the preparation of a stock solution of the pure and mixed system. Solvent stock solutions (i.e., aqueous NaCl and aqueous urea) were also prepared. Pure drug and surfactant stock solutions were prepared beyond their corresponding *cmc* value in an aqueous system or aqueous NaCl/aqueous urea solvent of the selected concentration. In the case of a mixed system, a prepared fixed concentration of IMP and TX-100 was mixed in various ratios at 298 K, and stock solution density was assumed to be constant in the case of the pure or mixed system.

### 2.2. Method

#### 2.2.1. Surface Tension Method 

**Figure 2:**
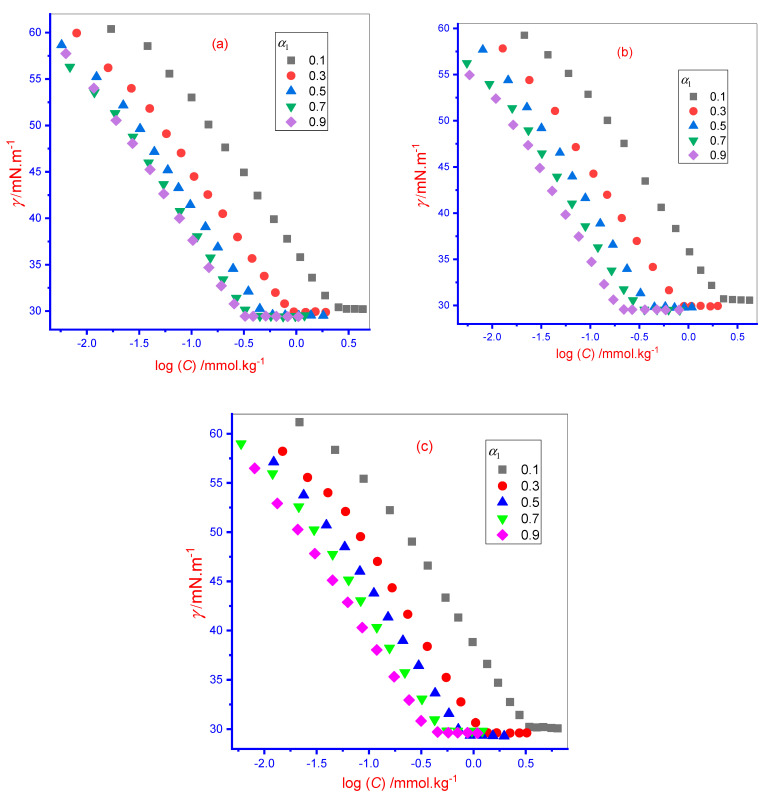
Graph of surface tension (*γ*) against concentration (*C*) plot for the IMP + TX-100 mixture in various mole fraction (α_1_) of TX-100 in (**a**) aqueous, (**b**) 0.050 mol∙kg^−1^ aqueous NaCl, and (**c**) 0.250 mol∙kg^−1^ aqueous U media.

A Sigma 701 (Drmstadt, Germany) Attension tensiometer was used to evaluate the surface tension (γ) of a singular component (IMP and TX-100) as well as the IMP + TX-100 mixture in three media through the means of the ring detachment process. A stock solution of 250 mmol·kg^−1^ of IMP was prepared for the determination of their *cmc* in aqueous and NaCl solvent, however, 300 mmol·kg^−1^ of IMP solution was prepared for the determination of *cmc* in the U solvent. For determination of *cmc* of pure TX-100 as well as IMP + TX-100 mixture, a stock solution of 12 mmol·kg^−1^ were prepared in all solvents. Various mole fractions of solution mixtures were prepared from pure IMP and TX-100 stock solutions in three media (aqueous/aqueous NaCl/aqueous U). The surface tension (*γ*) of singular components (IMP and TX-100) and the prepared different mole fraction of solution mixtures were measured through consecutive adding of concentrated prepared solution in aqueous/NaCl/U system at 298 K. The *γ* value reduced with every addition of solution in the aqueous/NaCl/U system up to a certain value and then became constant. Every reading of *γ* was taken three times and their mean value was considered as a final reading. After that, a graph was plotted between the attained *γ* of IMP/TX-100/IMP + TX-100 in the presence of different media versus log concentration (*C*) (log[IMP]/log[TX-100]/log[IMP + TX-100]). A representative plot of *γ* versus log[IMP + TX-100] in different media is shown in Figure 2. All obtained graphs showed a break spot called the *cmc* of the respective solution. The temperature error was found below ±0.20 K and the error in γ was found to be around ±0.20 mNm^−1^. It is clear from Figure 2 that no minima are shown nearby the *cmc* value, which indicates the purity of the employed compounds [1]. The error in *cmc* was found close to 3%.

#### 2.2.2. UV–Visible Study

To measure the UV–Visible absorbance spectra of the employed system, an Evolution 300 UV–Visible spectrometer (Thermo Scientific, Waltham, MA, USA) was used. First, the UV–visible absorbance spectra of IMP drug of appropriate concentration were noted, after that, the effect of increasing concertation of TX-100 on spectra of pure IMP was recorded in the aqueous system means absorbance spectra were recorded after each addition of TX-100. Deionized H_2_O was used throughout for the purpose of baseline correction. For UV–Visible measurements, the stock solution of IMP and TX-100 was prepared as 0.10 mmol·kg^−1^ and 10 mmol·kg^−1^, respectively.

## 3. Results and Discussion

### 3.1. cmc and Ideal cmc (cmc^id^) Values in Various Media

A tensiometry method was applied to evaluate the surface tension (*γ*) value of the solution of singular IMP, TX-100, and an IMP + TX-100 mixed system of five different ratios (i.e., five different mole fraction (*α*_1_) of TX-100 (TX-100 (0.1):IMP (0.9); TX-100 (0.3):IMP (0.7); TX-100 (0.5):IMP (0.5); TX-100 (0.7):IMP (0.3), and TX-100 (0.9):IMP (0.1)) to assess the *cmc* in various media (aqueous/0.050 mol·kg^−1^ aqueous NaCl/0.250 mol·kg^−1^ aqueous U) at 298 K. Throughout, the *α*_1_ and *α*_2_ = mole fraction of TX-100 and IMP, respectively, and *α*_1_ + *α*_2_ = 1. The surface tension (*γ*) value decreased linearly with the addition of a processed concentration (*C*) solution into the solvent until the pre-micellar region, and the lessening in *γ* value remained while waiting for the saturation of the air–solvent interfacial surface via added molecules, once the saturation of the interfacial surface occurred, the further added monomers started to accumulate amongst themselves to form associate structures called micelles [1]. Thus, the *γ* value did not change once touching a specific concentration of amphiphiles in solution, and this concentration of amphiphile was named the *cmc*. The acquired break point from the *γ* vs. log [*C*] report is shown in Figure 2 at 298 K (IMP + TX-100 mixture at different *α*_1_ of TX-100 in two media: (a) aqueous, and (b) 0.05 mol∙kg^−1^ aqueous NaCl. Obviously, the *γ* of singular and mixed amphiphiles solutions reduces rapidly through rising concentration (*C*) until the curve achieves a constant (almost) value at the concentration equivalent to *cmc* and continues at a nearly constant value, as displayed in Figure 2.

The obtained *cmc* value of the studied system (IMP, TX-100, and IMP + TX-100 in three media) is given in Table 1. In the aqueous solution, by the tensiometric method, the *cmc* value of singular IMP was found to be 41.85 mmol⋅kg^−1^ at 298 K, which is considerable in the acceptable agreement through earlier reported values (45.20 mmol⋅kg^−1^) [36]. A *cmc* value of singular TX-100 was attained of 0.31 mmol∙kg^−1^, also observing fine conformity with the literature (0.33 mmol⋅kg^−1^) [37]. The *cmc* value of the IMP drug was found to be much higher than the *cmc* value of TX-100. As the hydrophobic part of IMP is much smaller compared with the TX-100 hydrophobic part (see Figure 1), IMP starts the formation of micelles at a higher concentration compared with TX-100, as TX-100 dislocates the water structure to a larger level, which facilitates micelle formation at a lower concentration. Due to the much lower *cmc* value of TX-100, it shows much better surface as well as micellar properties [1]. Due to these advanced properties of TX-100, provoked us to explore the mixed micellization as well as the air–solvent surface behaviors of this surfactant through other less hydrophobic amphiphiles (such as amphiphilic ionic drugs).

Table 1 shows the variations in the *cmc* value of the IMP + TX-100 mixed system with the changes in the *α*_1_ of TX-100. The results showed that the *cmc* value of the IMP + TX-100 system reduced when the *α*_1_ of TX-100 enhanced in the solution. As specified in the literature, amphiphilic drug and surfactant mixtures formed mixed micelles via interaction through each other [33,38]. Here, the resulting *cmc* value of the mixtures showed that mixed micelle formation occurred at inferior concentrations due to the diminution in electrostatic repulsion among the constituent molecules in each used media variation. Table 1 also reveals that the *cmc* values of the mixtures were much lower compared to the *cmc* value of pure IMP and their value was achieved close to a *cmc* value of pure TX-100 at lower *α*_1_, and found below the *cmc* of pure TX-100 at the highest studied *α*_1_ (= 0.9) of TX-100, regardless of the employed solvent. The *cmc* of pure TX-100 was much lower compared to that of pure IMP, which means that TX-100 inclined to display aggregation behavior at much lower concentrations, therefore, the drug monomers only favorably pierced the micelles formed by TX-100 and helped in mixed micelle formations. Hence, it is understood from their obtained *cmc* that formed mixed micelles comprised mainly TX-100 constituents. The interaction between IMP and TX-100 mainly occurs due to ion–dipole interaction between the head group of both components and hydrophobic interaction between their chain during mixed micelle formation (Figure 1c).

The interaction between components (IMP and TX-100) in all media was computed by applying Clint’s theory [39]. Accordingly, for mixtures, the ideal *cmc* (*cmc*^id^) value of the mixed micelles was assessed by employing the following equation [39].

(1) $$\frac{1}{cmc^{\mathrm{id}}}=\frac{\alpha_{1}}{cmc_{1}}+\frac{\alpha_{2}}{cmc_{2}}$$

In the above equation, *cmc*_1_ = *cmc* value of constituent 1 (TX-100) and *cmc*_2_ = the *cmc* value of constituent 2 (IMP). In binary mixtures, the magnitude of nonideality was decided from the disparity amongst the value of experimental *cmc* and *cmc*^id^. As stated in the literature [1], synergistic interaction amongst components existed when *cmc*^id^ > *cmc*, antagonistic interaction was leading when *cmc*^id^ < *cmc*, whereas ideal mixing was observed if the experimental *cmc* was found equal to *cmc*^id^, which means interaction between the components and each other. Herein in our case, the condition *cmc*^id^ > *cmc* was attained in the mixed system, thereby suggesting nonideality irrespective of the different media used (Table 1). Therefore, the binary mixtures in our cases experienced primarily synergistic or attractive interactions. The considerable decline in the detected *cmc* value than the value of *cmc*^id^ was due to the increased hydrophobicity of the solution mixtures rife in the interactions amongst the ingredients irrespective of all media employed. The types of possible interaction between employed components were ion–dipole interactions amongst ionic and non-ionic hydrophilic parts; steric interactions amongst bulky parts of molecules; and van der Waals interactions amongst hydrophobic parts as well as H-bonding amongst monomers [1]. 

In aqueous sodium chloride (NaCl) media, the *cmc* value of IMP, TX-100 as well as their blended systems reduced more than the aqueous system (Table 1). The start of aggregation in aqueous NaCl media occurred at much lower concentrations compared to those noticed in the water due to enhanced interaction amongst the ingredients [40]. Overall, in aqueous NaCl media, the reduction in the *cmc* value of the singular substance as well as the mixed system was attributable to the screening enforcement put forth via the inorganic salt, which monitored the electrostatic repulsion amid the molecules of the ionic micelles, dipole interaction amid the monomers of the nonionic micelles, and ion–dipole interaction amongst ionic and non-ionic monomers of the mixed micelles. In aqueous salt media, the varying stability amongst the hydrophilic along with the hydrophobic interaction may possibly apply to the narrative alteration in the physical attributes of the solution’s components. A decrease in the *cmc* value may well be explained through the theory of counterion binding [1,41]. The added electrolyte counterions were attached through the hydrophilic portion of the components, obtaining a higher aggregation number (*N*_agg_) value and lower value of *cmc* [1]; this, consecutively, reduced the repulsive interactions among the head fractions of the components that arouses the start of the association at lower concentration [42]. Regardless of this, the add-on of salt to the pure and mixed system interrupted the hydration layer, causing the establishment of an expanded double-layer close to the hydrophilic portion. This ensuing falloff in the repulsive forces amongst molecules assisted the association process more extensively at reduced concentrations.

In contrast to the behavior of NaCl, in the existence of U, the value of *cmc* of the individual and mixed system generally enhanced, signifying that the formation of micelles and mixed micelles, respectively, are favored to start at a greater concentration compared with the aqueous solution. The occurrence of this behavior was accredited to fewer extensive interactions amongst the component monomers in aqueous U media [43]. The obtained higher *cmc* value here represented the familiar cosolvent consequence wielded by U. Herein, U enhanced the crashing aptitude of the iceberg structure adopted via the micellar solution, thus enhancing the solubility of the free monomers. Furthermore, amphiphilic molecules are extra stabilized in the U medium compared with the aqueous solution [44]. Through the adsorption of U on the exterior part of the charged/nonionic employed molecules, the reduction in hydrophobic interactions occurred, which caused the rise in additional electrostatic repulsive forces and in the *cmc* value. Herein, in the current study, the employed concentration of U (0.25 m·kg^−1^) was sufficient to stabilize the used constituents, thus postponing the molecular aggregations that occurred to some extent, which changed (increased) the *cmc* value of the IMP, TX-100, and IMP + TX-100 mixtures. Moreover, in accordance with a distinct outlook, it was considered that U precisely intermingled through the hydrophobic part of the molecules, lowering the molecules’ ability to play a part in hydrophobic binding, leading to the large value of *cmc* detected [45]. There was no reason for selecting 0.050 mol·kg^−1^ NaCl, and 0.250 mol·kg^−1^ U except to explore the impact of the salt and U on the interaction of IMP and TX-100. Additionally, our main objective had been to show how both constituents interacted in water as well as in NaCl/U media with the viewpoint of supplying further information for the essential and commonly used drug and surfactant mixtures in aqueous/non-aqueous media in drug delivery.

### 3.2. Mixed Micellization Behavior of IMP+TX-100 Mixed System 

The decrease in the *cmc* compared with the *cmc*^id^ value of the IMP+TX-100 mixture revealed nonideality, and the fact this took place was confirmation of synergism or attractive interactions amongst the compounds [1]. The model regarding the verdict of interaction amongst components of the mixed micelles was first given via Rubingh’s [46] and then on regular solution theory (RST), which allows for the valuation of the micellar content of TX-100 (first constituent) ($X_{1}^{\mathrm{Rb}}$). Equation (2) was resolved to obtain the $X_{1}^{\mathrm{Rb}}$ value.

(2) $$\frac{{\left(X_{1}^{\mathrm{Rb}}\right)}^{2}\ln\left[\left(\alpha_{1}cmc/X_{1}^{\mathrm{Rb}}cmc_{1}\right)\right]}{{\left(1-X_{1}^{\mathrm{Rb}}\right)}^{2}\ln\left[\left(1-\alpha_{1}\right)cmc/\left(1-X_{1}^{\mathrm{Rb}}\right)cmc_{2}\right]}=1$$

In the ideal condition, micellar composition of the TX-100 ingredient in the ideal system ($X_{1}^{\mathrm{id}}$) was evaluated by Motomura theory [47] using Equation (3).

(3) $$X_{1}^{\mathrm{id}}=\frac{\alpha_{1}cmc_{2}}{\alpha_{1}cmc_{2}+\alpha_{2}cmc_{1}}$$

For the entire system, the values of $X_{1}^{\mathrm{Rb}}$ and $X_{1}^{\mathrm{id}}$ of TX-100 achieved from Equations (2) and (3) are depicted in Table 1. The value of $X_{1}^{\mathrm{Rb}}$ attained was always greater than the employed *α*_1_ of TX-100, despite the value recorded at the *α*_1_ = 0.9 [48,49]. The achieved $X_{1}^{\mathrm{id}}$ value was found to be less than the $X_{1}^{\mathrm{id}}$ value at all *α*_1_ of TX-100 in all solvents, signifying that the involvement of the surfactant in mixed micelles was found to be less, as expected from ideal behavior, resulting in more IMP monomers taking part in mixed micelles than expected ideally, but much less than TX-100. The increase in $X_{1}^{\mathrm{id}}$ value was observed with an increase in *α*_1_ value in each media, but the value of $X_{1}^{\mathrm{Rb}}$ did not view any fixed trend with *α*_1_ in all media, however, there was an increase observed. Based on the results, we theorized that the formed mixed micelles were comprised of higher than 77% TX-100 and that the IMP simply intermingled with TX-100 micelles by penetrating in their micelles and forming mixed micelles, which advocated the enhancing of a hydrophobic atmosphere. In the aqueous salt or aqueous U solvent, the $X_{1}^{\mathrm{Rb}}$ value did not show any trend with an increase in *α*_1_ in the IMP + TX-100 mixed system, however, the average value of $X_{1}^{\mathrm{Rb}}$ in a different solvent were attained in the subsequent order: $X_{1}^{\mathrm{Rb}}$ (NaCl) > $X_{1}^{\mathrm{Rb}}$ (aqueous system) > $X_{1}^{\mathrm{Rb}}$ (U) (Table 1).

The attained value of $X_{1}^{\mathrm{Rb}}$ by means of Equation (2) was further applied to determine the degree of interaction (interaction parameter ($\beta^{\mathrm{Rb}}$)) amongst the utilized ingredients in the mixed system (IMP + TX-100). The value of $\beta^{\mathrm{Rb}}$ was assessed through operating Equation (4) [1,46].

(4) $$\beta^{\mathrm{Rb}}=\frac{\ln(cmc\alpha_{1}/cmc_{1}X_{1}^{\mathrm{Rb}})}{{\left(1-X_{1}^{\mathrm{Rb}}\right)}^{2}}$$

The $\beta^{\mathrm{Rb}}$ value informs the extent of interaction (attractive) between thee amphiphiles along with the deviation of the micellar real mixed system with ideal behavior. According to this theory (Rubinghs [46]), the *β*^Rb^ should be constant for a binary mixed micellar system within the change of mole fraction of the component. However, in most cases, the $\beta^{\mathrm{Rb}}$ value does not remain constant despite the variations in *α*_1_ due to the limitation of this model. The entire attained values of *β*^Rb^ of the IMP + TX-100 mixture in all media are presented in Table 1. Regarding the value of *β*^Rb^, three possibilities are reported in the literature [1]: (i) *β*^Rb^ value close to zero for the case of ideal mixed system; (ii) *β*^Rb^ > 0 for solution mixtures categorized via repulsive interactions; and (iii) *β*^Rb^ < 0 for the mixed system wherein principally attractive interactions amongst the constituents are detected [1]. Table 1 shows that all obtained *β*^Rb^ values in our case were negative, denoting that synergistic or attractive interactions occurred during mixed micelle formation and the *β*^Rb^ value increased through an increase in *α*_1_ with one exception at *α*_1_ = 0.3 in the presence of NaCl. The obtained *β*^Rb^ value for IMP + TX-100 mixtures were between −1 and −8 in all of the studied media (Table 1), while for the mixed micelle formation, generally the interaction amongst components is either attractive interactions or synergism. The synergism is ordinarily settled in whichever mixtures the subsequent two requirements are accomplished: (a) $\beta^{\mathrm{Rb}}$ < zero, and (b) $\left|\;\beta^{\mathrm{Rb}}\right|$ > $\left|ln(cmc_{1}/cmc_{2})\right|$. If only the first term is present, then attractive interaction is noticed. In our case, at a lower *α*_1_ value (i.e., *α*_1_ = 0.1, 0.3, and 0.5), only the first condition was attained, therefore, attractive interactions existed at *α*_1_ = 0.1, 0.3, and 0.5. However, at the higher *α*_1_ of TX-100 (*α*_1_ = 0.7, and 0.9), an indication of synergism was observed because both terms were fulfilled at higher *α*_1_ of TX-100. The reason for the achieved $\beta^{\mathrm{Rb}}$ negative value can be attributed to the hydrophobic interactions among the hydrophobic part of IMP and TX-100 [49]. The interaction amongst components grew from the ion–dipole interactions amongst the polar fraction of the surfactant and the positively charged head group of IMP along with the interaction due to H-bonding was also detected amongst the hydroxyl groups of the TX-100 surfactant and cationic IMP drug. Moreover, cation–π interactions occurred amongst the cyclic rings of employed constituents [50]. The *β*^Rb^ value of the IMP + TX-100 + NaCl mixture was predictable to be additionally negative when compared to that obtained for the IMP + TX-100 + H_2_O mixtures and their values for IMP + TX-100 + U mixtures were expected to be less negative compared with the aqueous system, but this was not detected practically (Table 1).

The obtained $X_{1}^{\mathrm{Rb}}$ and $\beta^{\mathrm{Rb}}$ values were further used to evaluate values of the activity coefficients ($f_{1}^{\mathrm{Rb}}$ (TX-100) and $f_{2}^{\mathrm{Rb}}$ (IMP)) of amphiphiles using the subsequent equations.

(5) $$f_{1}^{\mathrm{Rb}}=exp[\beta^{\mathrm{Rb}}\left(1-X_{1}^{\mathrm{Rb}}{)}^{2}\right]$$

(6) $$f_{2}^{\mathrm{Rb}}=exp[\beta^{\mathrm{Rb}}\left(X_{1}^{\mathrm{Rb}}{)}^{2}\right]$$

The $f_{1}^{\mathrm{Rb}}$ and $f_{2}^{\mathrm{Rb}}$ values are shown in Table 2. In all cases, both component activity coefficient values were achieved under unity, showing positive interactions amongst IMP and TX-100, and the mixed micellar solution also showed non-ideal behavior in various media. In each case, the $f_{1}^{\mathrm{Rb}}$ value was found to be higher than the $f_{2}^{\mathrm{Rb}}$ value, confirming that mixed micelles include a higher proportion of TX-100 compared with IMP. Moreover, through an increase in the *α*_1_, the $f_{2}^{\mathrm{Rb}}$ value decreased, revealing that the contribution of IMP in mixed micelles decreased through the surge of *α*_1_ (Table 2). The values of $f_{1}^{\mathrm{Rb}}$ and $f_{2}^{\mathrm{Rb}}$ displayed no dependence on the nature of the solvent employed.

### 3.3. Thermodynamic Parameter 

The proclivity of amphiphilic molecules to self-aggregates can also be used to evaluate the different thermodynamic parameters. One of the thermodynamic parameters, known as Gibbs free energy ($∆G_{m}^{o}$), was assessed for the association of singular components (IMP and TX-100) along with their blended system in different ratios using Equation (7) [51,52,53].

(7) $$∆G_{m}^{o}=RT\ln X_{cmc}$$

**Figure 3:**
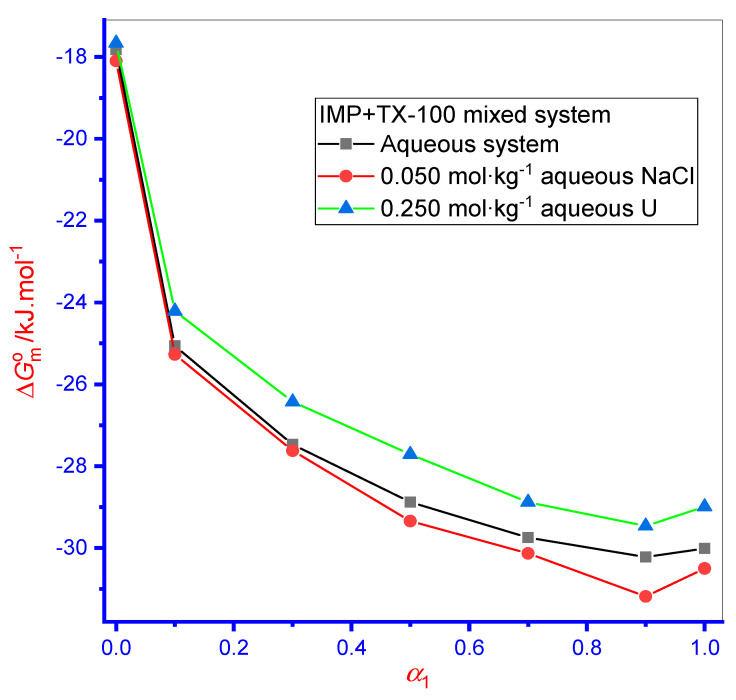
Variation in $∆G_{m}^{o}$ value with the change in mole fraction (*α*_1_) of TX-100 in different media. Relative standard uncertainties (*u_r_*) is *u_r_*($∆G_{m}^{o}$ ) = ±3%.

In Equation (7), *X_cmc_* symbolizes the *cmc* in mole fractions. Here, the value of $∆G_{m}^{o}$ attained for each system of all media was negative, showing that pure/mixed association was a spontaneous process (Figure 3). Higher negative value of $∆G_{m}^{o}$ was found for the case of mixture (IMP + TX-100) micellization than for the $∆G_{m}^{o}$ value of the pure IMP micellization phenomena, demonstrating that spontaneity was greater in binary solution mixtures. The $∆G_{m}^{o}$ value of pure TX-100 was attained in a higher magnitude than the $∆G_{m}^{o}$ value of pure IMP, showing that TX-100 had more spontaneity as well as hydrophobicity than IMP (Figure 3). Consequently, the association was smoothed in TX-100 than in IMP. Additionally, with enhanced *α*_1_ in mixed systems, an increase in the negative $∆G_{m}^{o}$ value occurred, thus endorsing the spontaneity of the mixtures through an increase in *α*_1_ value and at the highest *α*_1_, their values were beyond the $∆G_{m}^{o}$ value of both components (Figure 3). In the aqueous solution, the $∆G_{m}^{o}$ value for singular IMP was −17.82 kJ·mol^–1^, which well agreed with an earlier report (−17.90 kJ·mol^–1^) [54]. Additionally, the $∆G_{m}^{o}\;$value by singular TX-100 was also found to be similar to the previously reported value [55]. 

The $∆G_{m}^{o}$ became increasingly negative for singular and binary mixtures in the aqueous NaCl media compared with the aqueous system. Hence, micellization initiated at an inferior concentration in the aqueous NaCl media, a connotation that the process also becomes spontaneous (Figure 3). However, the negative value of $∆G_{m}^{o}$ for pure and mixed system was reduced in the U medium, which revealed delayed association to some extent and decreased spontaneity compared with the aqueous medium. 

Excess free energy ($\Delta G_{\mathrm{ex}}^{m}$ (mixed micelles)) for the current system is another thermodynamic parameter that can be calculated by the subsequent equations [56,57,58].

(8) $$∆G_{\mathrm{ex}}^{m}=RT\left[X_{1}^{\mathrm{Rb}}\ln f_{1}^{\mathrm{Rb}}+\left(1-X_{1}^{\mathrm{Rb}}\right)\ln f_{2}^{\mathrm{Rb}}\right]$$

The $∆G_{\mathrm{ex}}^{m}$ values computed for the IMP + TX-100 mixed system in various media are depicted in Table 2. The $∆G_{\mathrm{ex}}^{m}$ value attained for the mixed micelles was negative, which veiled the raised stability of both mixed micelles corresponding to their individual component’s micelles. Generally, with an enhanced of *α*_1_, the $\Delta G_{\mathrm{ex}}^{m}\;$value was attained to increase with the exception in some *α*_1_ also specifying that stability of mixed system increased with *α*_1_.

The thermodynamic stability of IMP+TX-100 mixtures mixed micelles can also be calculated through the Maeda model [59]. By this method, the thermodynamic stability in the form of Gibb’s free energy of association ($\Delta G_{\mathrm{Maeda}}^{0}$) which was stand on the phase separation model is evaluated by following equation [59].

(9) $$\Delta G_{\mathrm{Maeda}}^{0}=RT\;(B_{0}+B_{1}X_{2}^{\mathrm{Rb}}+B_{2}\left(X_{2}^{\mathrm{Rb}}{)}^{2}\right)$$

In the above equation, terms *B*_0_, *B*_1_, and *B*_2_ are self-governing, and their values can be defined through the subsequent relationships [59,60]:

*B*_0_ = ln *cmc*_1_ (10)

*B*_2_ = −*β*^m^ (11)

(12) $$\ln\frac{cmc_{2}}{cmc_{1}}=B_{1}+B_{2}\;$$

where *β*^m^ is the interaction parameter and their values were evaluated using Equation (4). The Maeda model is valid when one of the ingredients in the binary mixtures is non-ionic in nature as the stability of the ingredients in mixed micelles are principally reliant on (a) the interactions amongst the head groups as well as (b) the interactions amongst the hydrophobic portions. Fewer negative *β*^m^ values and positive *B*_1_ values revealed the repulsive interaction amongst the head groups. In reverse, a negative value of *B*_1_ showed hydrocarbon part interactions amongst the ingredients was greater, which had the main effects on the mixed micellar systems’ stability. This suggests that less transformation of the ionic constituents occurred into micellar form from their monomeric form, which means that the non-ionic ingredient was dominant in the formed mixed micelles. Herein, *B*_1_ was found to be positive at lower *α*_1_ of TX-100 (0.1, 0.3, and 0.5) and negative at higher *α*_1_ of TX-100 (0.7 and 0.9), showing that at lower *α*_1_ repulsive forces amongst the head group–head group prevailed in the mixture, due to which the formation of the mixed micelle was deferred to some level, which means that it occurred at elevated concentration. Negative *B*_1_ value showed that the chain–chain interactions amongst the employed components dominated for the stability of the formed mixed micelles. However, the obtained *B*_1_ value is not given in the manuscript. From the structure of IMP and TX-100, it can be found that their chain lengths were unlike, and chain–chain interactions assisted in stabilizing the mixed micellar solutions. 

Table 2 showed that the value of $\Delta G_{\mathrm{Maeda}}^{0}$ in our cases were negative and their value enhanced via the increase in the *α*_1_ value of surfactant [54]. This showed that the stability of mixed micelles was enhanced via an increase in the *α*_1_ value of the surfactant. Table 2 also shows that in aqueous NaCl solvent, the negative value of $\Delta G_{\mathrm{Maeda}}^{0}$ increased for all *α*_1_ of TX-100. This phenomenon again proved that the stability of the mixed micelles improved in the NaCl system rather than the aqueous system because the effect of added NaCl in the micellar solution was based on the fact of “salting-out”, electrostatic screening, along with lessened steric effects. On the other hand, in the aqueous U solvent, the negative value of $\Delta G_{\mathrm{Maeda}}^{0}$ observed was low for all *α*_1_ (Table 2). Urea destabilized the mixed micellar solution due to the increase in repulsive interactions amongst the constituent’s head groups. It was also shown that the $\Delta G_{m}^{0}$ and $\Delta G_{\mathrm{Maeda}}^{0}$ value differed greatly, which was supposed to be induced via the counterion fraction that occurred close to the mixed micelles. Therefore, as specified via the Maeda model, if the chain lengths of both ingredients are dissimilar, then interactions among the chain–chain take part in the stability of the mixed micelle.

### 3.4. UV–Visible Study

**Figure 4:**
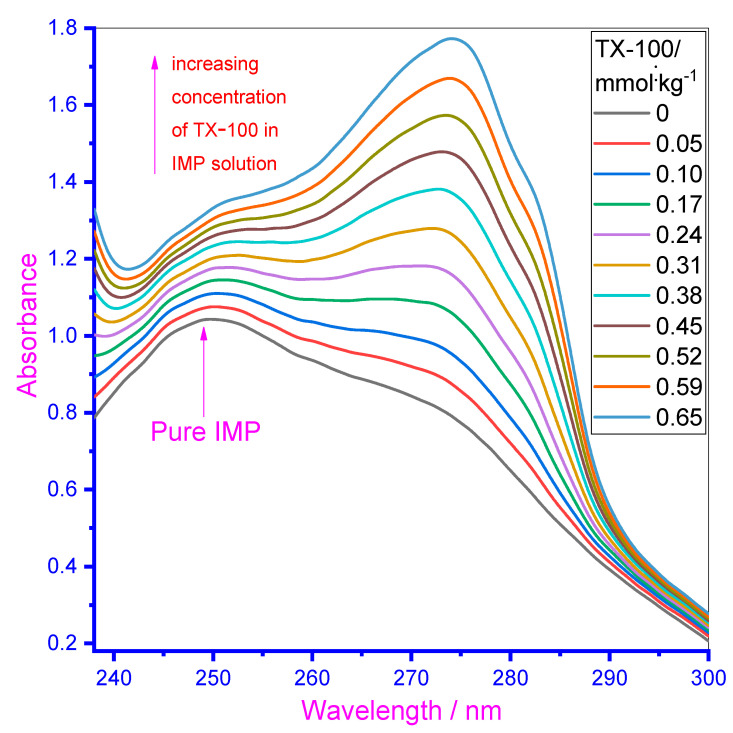
UV–Visible spectra of the drug in the absence along with the existence of a growing concentration of TX-100.

To further investigate the molecular interaction of the employed drug IMP with TX-100, another spectroscopic method—UV–visible was used [61]. The aromatic ring of the IMP drug is liable for their considerable absorption characteristic. The absorption spectra of the drug were noted through increasing TX-100 concentration and the obtained results are given in Figure 4 for an aqueous system. The solution of TX-100 was prepared in the presence of 0.10 mmol·kg^−1^ IMP to avoid a dilution effect during titration. The spectra of IMP (0.10 mmol·kg^−1^) depicted one clear maximum absorption wavelength (λ_max_) at 249 nm in the aqueous system, which was ascribed to π–π* transition. Effect of increasing the TX-100 concentration is noted on the absorption spectra of IMP of fixed concentration (concentration of TX-100 varies from 0.05 mmol·kg^−1^ to 0.65 mmol·kg^−1^ in IMP solutions) (Figure 4). With the addition of TX-100 in the solution of IMP, the absorption strength of drug enhanced (i.e., hyperchromic effect occurred due to the H-bonding and electrostatic interaction) between the employed components [22,62]. At a lower concentration of TX-100, that the spectral peak of IMP (249 nm) shifted toward the higher wavelength means that in the mixtures, red shifting occurred, authorizing the interaction between IMP and the TX-100 mixture. Some of the initially added concentration of TX-100 into the IMP solution exist in their monomer form and after further addition of TX-100 into the solution of IMP, TX-100 monomeric forms turn into the micellar form and the initially obtained peak (near to 249 nm) disappeared due to complex formation between the components. A new peak appeared at a higher wavelength (above 275 nm) and showed higher wavelengths with the addition of more TX-100. At higher concentrations of the surfactant, the intercalation of IMP molecules inside the palisade layer of the surfactant micelles took place and possibly a new peak (above 275 nm) mainly occurred due to TX-100 [63]. Therefore, the overall results of titration specify the strong interaction of IMP with TX-100 as the absorption increased with an increase in TX-100 concentration. Red shift (more than 25 nm) was also detected (obtained redshift showed a rise in the wavelength and related reduction in the frequency along with photon energy of electromagnetic emission), due to the IMP–TX-100 complex formation. Attraction between IMP and TX-100 could also be due to no charge being present on the head group of non-ionic TX-100, attracting positively charged IMP drug monomers. Therefore, complexation was detected between IMP and TX-100.

Herein, for quantitative assessment of the surfactant TX-100 binding or interaction with the IMP drug, the attained absorbance value was utilized in the equation of Benesi–Hildebrand [64].

(13) $$\frac{1}{A-A_{0}}=\frac{1}{K\left(A_{max}-A_{0}\right){\left[\mathrm{TX}-100\right]}^{2}}+\frac{1}{A_{max}-A_{0}}$$

**Figure 5:**
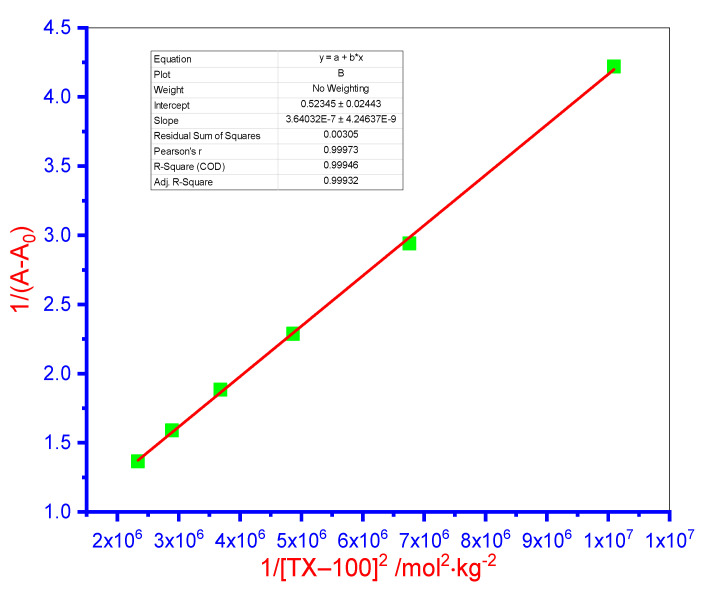
The plot of 1/(A − A_0_) against 1/[TX-100]^2^ for the interaction of the IMP drug with TX-100.

In Equation (13), symbols *K* and *A*_0_ depict the binding constant and IMP absorbance value in pure form, respectively. *A* and *A_max_* showed the IMP absorbance value in the presence and infinite concentration of TX-100, correspondingly. The $\frac{1}{A-A_{0}}$ vs. 1/[TX-100]^2^ plot displayed the straight line, which validates the 1 (drug):2 (TX-100) complex formation and their achieved plot was graphically displayed in Figure 5. On the other hand, the Benesi–Hildebrand plot assuming a 1:1 stoichiometry gave rise to a clearly curvilinear fit, indicating that this is an incorrect hypothesis and therefore, the actual stoichiometric ratio for complexes of IMP–TX-100 is principally 1:2 [62].

In the current study, the *K* value (attained from the ratio of intercept/slope) of IMP + TX-100 complex was attained as 1.40 × 10^6^ mol^−2^·kg^2^. In Figure 5, the value of the correlation coefficient acquired of 0.9995 recommends the excellent linear fit. The evaluated *K* value of the IMP + TX-100 complex was further exploited to assess the value of free energy change (Δ*G*) using Equation (14) [65].

(14) ΔG=−RTlnK

From Equation (14), the attained Δ*G* value was found to be −35.14 kJ·mol^−1^, which showed that the IMP + TX-100 complex formation is thermodynamically favorable (negative Δ*G*) (i.e., the reaction is spontaneous) [66]. The acquired negative Δ*G* value illustrates the decrease in self-electrostatic repulsion amongst the head group of molecules, which revealed desirable binding between IMP and TX-100.

## 4. Conclusions

Non-ionic surfactants are suitable for drug delivery and are a very auspicious group of penetration enhancers; therefore, the current analysis is valued in terms of the awareness of the interactions of the drug and surfactant. Herein, the interaction between imipramine hydrochloride (IMP) and TX-100 mixtures were investigated in detail by the tensiometric method in five different ratios in three solvents (aqueous/aqueous NaCl/aqueous U). The interaction between the IMP + TX-100 mixture in the aqueous system was also evaluated by means of UV–Visible study. Tensiometric measurements showed that the IMP and TX-100 mixtures in aqueous and other employed solvents go through numerous physicochemical variations due to several interactions amongst components, and therefore, the mixtures showed improved micellar and surface assets compared with the singular IMP or TX-100. The entire mixed system (IMP + TX-100) provided a lower *cmc* value compared with ideal *cmc* values along with negative $\beta^{\mathrm{Rb}}$ values signifying attractive attraction or synergism in the mixed system (IMP + TX-100). The negative $\Delta G_{\mathrm{mic}}^{\circ }\;$values indicate that the micellization process was spontaneous phenomena, while the obtained negative $\Delta G_{\mathrm{ex}}^{m}$ values confirmed the stability of mixed micelles of the IMP and TX-100 mixture. That the obtained activity coefficients showed deviation from unity specifies that the mixed systems act nonideally. The outcomes suggest that non-ionic surfactants will possibly assist as a skilled drug delivery agent and enlighten the bioavailability of the drug.

## Figures and Tables

**Table 1:** Various physicochemical parameters for IMP+TX-100 mixture in several solvents at 298 K ^a^.

α_1_	*cmc* (mmol⋅kg^−1^)	*cmc*^id^ (mmol⋅kg^−1^)	$X_{1}^{\mathrm{Rb}}$	$X_{1}^{\mathrm{id}}$	$\beta^{\mathrm{Rb}}$	ln(*cmc*_1_/*cmc*_2_)
Aqueous solution						
0	41.85					
0.1	2.25	2.86	0.8029	0.9384	−2.18	
0.3	0.85	0.99	0.8745	0.9833	−2.85	
0.5	0.48	0.61	0.8585	0.9928	−4.35	−4.92
0.7	0.34	0.43	0.8633	0.9969	−5.41	
0.9	0.28	0.34	0.8913	0.9992	−6.42	
1	0.31					
0.050 mol∙kg^−1^ aqueous NaCl						
0	37.25					
0.1	2.06	2.36	0.8518	0.9430	−1.51	
0.3	0.80	0.82	0.9615	0.9846	−1.05	
0.5	0.40	0.50	0.8652	0.9933	−4.31	−5.0
0.7	0.29	0.36	0.8774	0.9971	−5.15	
0.9	0.19	0.28	0.8411	0.9993	−8.19	
1	0.25					
0.250 mol∙kg^−1^ aqueous U						
0	44.64					
0.1	3.17	4.21	0.7712	0.9151	−2.15	
0.3	1.29	1.50	0.8740	0.9765	−2.40	
0.5	0.77	0.91	0.8796	0.9898	−3.43	−4.58
0.7	0.48	0.65	0.8406	0.9956	−5.53	
0.9	0.38	0.51	0.8575	0.9989	−7.03	
1	0.46					

^a^ Relative standard uncertainties (*u_r_*) are *u_r_*(*cmc/cmc*^id^) = ±3%, *u_r_*($X_{1}^{\mathrm{Rub}}$/$X_{1}^{\mathrm{id}}$) = ±3%, and *u_r_*($\beta^{\mathrm{Rb}}$) = ±3%.

**Table 2:** Thermodynamic parameters and activity coefficient for IMP + TX-100 mixture in several solvents at 298 K ^a^.

α_1_	$\Delta G_{\mathrm{Maeda}}^{0}\;(\mathrm{kJ}\cdot \mathrm{mol}{-}^{1})$	$\Delta G_{\mathrm{ex}}^{m}\;(\mathrm{kJ}\cdot \mathrm{mol}{-}^{1})\;$	$\;\;\;\;\;\;\;\;\;\;\;\;\;f_{1}^{\mathrm{Rb}}$	$\;\;\;\;\;\;\;\;\;f_{2}^{\mathrm{Rb}}$
Aqueous system				
0.1	−18.51	−0.85	0.9188	0.2453
0.3	−19.30	−0.78	0.9560	0.1128
0.5	−19.64	−1.31	0.9166	0.0405
0.7	−19.97	−1.58	0.9039	0.0178
0.9	−20.27	−1.54	0.9270	0.0061
0.050 mol∙kg^−1^ aqueous NaCl				
0.1	−19.18	−0.47	0.9674	0.3342
0.3	−20.17	−0.10	0.9984	0.3777
0.5	−20.12	−1.25	0.9246	0.0397
0.7	−20.40	−1.37	0.9255	0.0189
0.9	−21.29	−2.71	0.8132	0.0030
1				
0.250 mol∙kg^−1^ aqueous U				
0.1	−17.38	−0.94	0.8936	0.2785
0.3	−18.27	−0.66	0.9626	0.1597
0.5	−18.57	−0.90	0.9515	0.0705
0.7	−19.07	−1.84	0.8689	0.0201
0.9	−19.55	−2.13	0.8670	0.0057

^a^ Relative standard uncertainties (*u_r_*) are *u_r_*($\Delta G_{\mathrm{Maeda}}^{o}$) = ±4%, *u_r_*($\Delta G_{\mathrm{ex}}^{m}$) = ±5%, and *u_r_*($f_{1}^{\mathrm{Rb}}/f_{2}^{\mathrm{Rb}}$) = ±3%.

## Data Availability

All relevant data are within the paper.
